# Di-μ_2_-chlorido-bis­[chlorido(η^5^-2,3,4,5-tetra­methyl-1-propyl­cyclo­penta­dien­yl)iridium(III)]

**DOI:** 10.1107/S1600536813005072

**Published:** 2013-02-28

**Authors:** Joseph S. Merola, David Morris, Nicholas De Weerd

**Affiliations:** a Department of Chemistry 0212, Virginia Tech, Blacksburg, VA 24061, USA

## Abstract

The asymmetric unit of the title complex, [Ir_2_Cl_4_(C_12_H_19_)_2_], a versatile starting material for the preparation of uniquely substituted penta­alkyl­cyclo­penta­dien­yl–iridium complexes, consists of an iridium(III) atom, a substituted cyclo­penta­dienyl ligand and two chlorine ligands. The full dimer is generated by an inversion center. In the dimer, the two Ir^III^ atoms and two bridging Cl atoms form a perfectly planar ring. The two Ir^III^ atoms and the two terminal Cl atoms also form a rigorous plane that is orthogonal [89.48 (3)°] to the Ir_2_Cl_2_ ring. The plane of the cyclo­penta­dienyl ligand forms a dihedral angle of 54.06 (7)° with respect to the Ir_2_Cl_2_ ring.

## Related literature

For the structure of the analogous penta­methyl­cyclo­penta­dienyl compound (CCDC 508943), see: Churchill & Julius (1977 ▶). For the structure of the 1-phenyl-2,3,4,5-tetra­methyl­cyclo­penta­­dienyl complex (CCDC 802289), see: Liu *et al.* (2011 ▶).
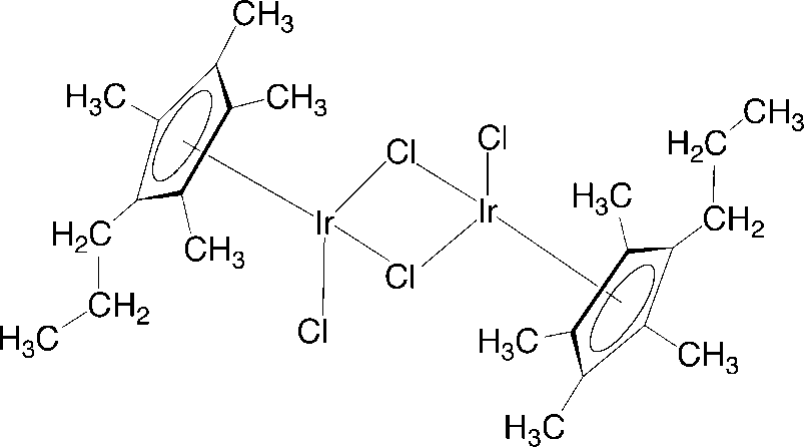


## Experimental

### 

#### Crystal data

[Ir_2_Cl_4_(C_12_H_19_)_2_]
*M* *_r_* = 852.74
Monoclinic, 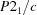

*a* = 8.84367 (12) Å
*b* = 8.83900 (12) Å
*c* = 17.2662 (2) Å
β = 103.6737 (14)°
*V* = 1311.43 (3) Å^3^
*Z* = 2
Mo *K*α radiation
μ = 10.56 mm^−1^
*T* = 100 K
0.26 × 0.12 × 0.05 mm

#### Data collection

Agilent Xcalibur (Eos, Gemini ultra) diffractometer
Absorption correction: Gaussian (*CrysAlis PRO*; Agilent, 2011 ▶) *T*_min_ = 0.225, *T*_max_ = 0.647
20544 measured reflections
4440 independent reflections
3968 reflections with *I* > 2σ(*I*)
*R*_int_ = 0.043

#### Refinement

*R*[*F* ^2^ > 2σ(*F* ^2^)] = 0.019
*wR*(*F* ^2^) = 0.040
*S* = 1.06
4440 reflections
141 parameters
H-atom parameters constrained
Δρ_max_ = 1.02 e Å^−3^
Δρ_min_ = −1.18 e Å^−3^

### 

Data collection: *CrysAlis PRO* (Agilent, 2011 ▶); cell refinement: *CrysAlis PRO*; data reduction: *CrysAlis PRO*; program(s) used to solve structure: *SHELXS97* (Sheldrick, 2008 ▶); program(s) used to refine structure: *SHELXL97* (Sheldrick, 2008 ▶); molecular graphics: *OLEX2* (Dolomanov *et al.*, 2009 ▶); software used to prepare material for publication: *OLEX2*.

## Supplementary Material

Crystal structure: contains datablock(s) I, global. DOI: 10.1107/S1600536813005072/pk2466sup1.cif

Structure factors: contains datablock(s) I. DOI: 10.1107/S1600536813005072/pk2466Isup2.hkl

Supplementary material file. DOI: 10.1107/S1600536813005072/pk2466Isup3.cml

Additional supplementary materials:  crystallographic information; 3D view; checkCIF report

## Figures and Tables

**Fig. 1.**
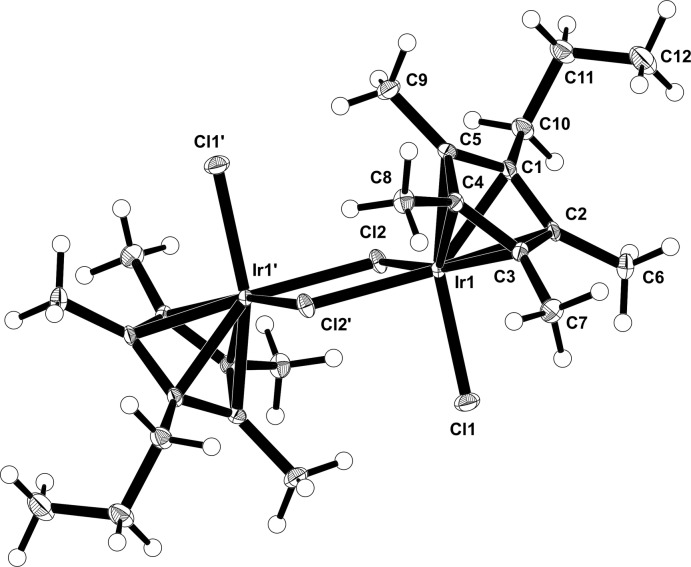
Thermal ellipsoid plot of the title compound. Ellipsoids are displayed at the 50% probability level. The entire molecule is generated by the inversion center and atoms related by inversion [-x, 2-y, 1-z] are labelled with (') after the atom designator. Only carbon atoms for one asymmetric unit are labelled.

## supplementary crystallographic information

### Comment

Di-µ_2_-chlorido-bis[chlorido(η^5^-2,3,4,5-tetramethyl-1-propylcyclopentadienyl)iridium(III)] is a useful starting material for half-sandwich complexes of iridium. Compared with the pentamethycylopentadienyl variety, complexes of the propyl-tetramethyl complexes are more soluble in organic solvents. Structurally, the core of the title compound is superimposable with the parent pentamethylcyclopentadiene complex.

The unit-cell dimensions for the pentamethylcyclopentadienyl compound (Churchill, *et al.* (1977) are quite similar with the exception of the *c* axis being 1.5 A longer due to the longer chain on the cyclopentadienyl ligand.

### Experimental

Iridium(III)chloride hydrate was purchased from Pressure Chemical Company. 1-Propyl-2,3,4,5-tetramethyl cyclopentadiene was purchased from Sigma-Aldrich. The iridium(III)chloride hydrate (1.00 g, 2.84 mmol) was dissolved in 100 ml methanol. 1-Propyl-2,3,4,5-tetramethyl cyclopentadiene (0.70 g,4.25 mmol) was added and the mixture refluxed under nitrogen for 48 hrs. The methanol was removed under vacuum, the solid dissolved in dichloromethane and precipitated with diethylether to yield crystals of the title material. A suitable single-crystal was chosen from those that formed.

### Refinement

Hydrogen atoms were treated with a riding model with C—H distances of 0.99 Å (methylene) and 0.98 Å (methyl). U_iso_(H) values were set to either 1.2U_eq_ or 1.5U_eq_ (methyl only) of the attached atom.

### Crystal data

**Table:**

[Ir_2_Cl_4_(C_12_H_19_)_2_]	*F*(000) = 808
*M**_r_* = 852.74	*D*_x_ = 2.159 Mg m^−^^3^
Monoclinic, *P*2_1_/*c*	Mo *K*α radiation, λ = 0.7107 Å
*a* = 8.84367 (12) Å	Cell parameters from 11826 reflections
*b* = 8.83900 (12) Å	θ = 3.6–32.4°
*c* = 17.2662 (2) Å	µ = 10.56 mm^−^^1^
β = 103.6737 (14)°	*T* = 100 K
*V* = 1311.43 (3) Å^3^	Irregular, red
*Z* = 2	0.26 × 0.12 × 0.05 mm

### Data collection

**Table:**

Agilent Xcalibur (Eos, Gemini ultra) diffractometer	4440 independent reflections
Radiation source: Enhance (Mo) X-ray Source	3968 reflections with *I* > 2σ(*I*)
Graphite monochromator	*R*_int_ = 0.043
Detector resolution: 16.0122 pixels mm^-1^	θ_max_ = 32.5°, θ_min_ = 3.7°
ω scans	*h* = −13→13
Absorption correction: gaussian (*CrysAlis PRO*; Agilent, 2011)	*k* = −13→13
*T*_min_ = 0.225, *T*_max_ = 0.647	*l* = −25→25
20544 measured reflections	

### Refinement

**Table:**

Refinement on *F*^2^	Primary atom site location: structure-invariant direct methods
Least-squares matrix: full	Secondary atom site location: difference Fourier map
*R*[*F*^2^ > 2σ(*F*^2^)] = 0.019	Hydrogen site location: inferred from neighbouring sites
*wR*(*F*^2^) = 0.040	H-atom parameters constrained
*S* = 1.06	*w* = 1/[σ^2^(*F*_o_^2^) + (0.0107*P*)^2^ + 0.0112*P*] where *P* = (*F*_o_^2^ + 2*F*_c_^2^)/3
4440 reflections	(Δ/σ)_max_ = 0.003
141 parameters	Δρ_max_ = 1.02 e Å^−^^3^
0 restraints	Δρ_min_ = −1.18 e Å^−^^3^

### Special details

**Table:**

Geometry. All e.s.d.'s (except the e.s.d. in the dihedral angle between two l.s. planes) are estimated using the full covariance matrix. The cell e.s.d.'s are taken into account individually in the estimation of e.s.d.'s in distances, angles and torsion angles; correlations between e.s.d.'s in cell parameters are only used when they are defined by crystal symmetry. An approximate (isotropic) treatment of cell e.s.d.'s is used for estimating e.s.d.'s involving l.s. planes.
Refinement. Refinement of *F*^2^ against ALL reflections. The weighted *R*-factor *wR* and goodness of fit *S* are based on *F*^2^, conventional *R*-factors *R* are based on *F*, with *F* set to zero for negative *F*^2^. The threshold expression of *F*^2^ > σ(*F*^2^) is used only for calculating *R*-factors(gt) *etc*. and is not relevant to the choice of reflections for refinement. *R*-factors based on *F*^2^ are statistically about twice as large as those based on *F*, and *R*- factors based on ALL data will be even larger.

### Fractional atomic coordinates and isotropic or equivalent isotropic displacement parameters (Å^2^)

**Table:**

	*x*	*y*	*z*	*U*_iso_*/*U*_eq_	
Ir1	0.648925 (10)	0.848148 (9)	0.034000 (5)	0.00818 (3)	
Cl1	0.61998 (8)	0.77491 (7)	−0.10212 (4)	0.01940 (13)	
Cl2	0.36739 (7)	0.88743 (6)	0.00829 (4)	0.01276 (11)	
C1	0.8565 (3)	0.8778 (3)	0.12571 (14)	0.0114 (4)	
C2	0.8698 (3)	0.7453 (3)	0.07978 (14)	0.0107 (4)	
C3	0.7466 (3)	0.6410 (2)	0.08763 (14)	0.0107 (4)	
C4	0.6597 (3)	0.7121 (3)	0.13820 (14)	0.0111 (4)	
C5	0.7257 (3)	0.8590 (3)	0.16167 (14)	0.0114 (4)	
C6	0.9915 (3)	0.7111 (3)	0.03555 (16)	0.0169 (5)	
H6A	0.9426	0.6665	−0.0163	0.025*	
H6B	1.0669	0.6397	0.0664	0.025*	
H6C	1.0449	0.8048	0.0274	0.025*	
C7	0.7237 (3)	0.4861 (3)	0.05290 (15)	0.0164 (5)	
H7A	0.6226	0.4468	0.0575	0.025*	
H7B	0.8067	0.4195	0.0818	0.025*	
H7C	0.7269	0.4901	−0.0034	0.025*	
C8	0.5254 (3)	0.6462 (3)	0.16461 (16)	0.0158 (5)	
H8A	0.4363	0.7149	0.1503	0.024*	
H8B	0.5538	0.6318	0.2225	0.024*	
H8C	0.4978	0.5483	0.1384	0.024*	
C9	0.6723 (3)	0.9646 (3)	0.21693 (15)	0.0186 (5)	
H9A	0.6986	1.0687	0.2055	0.028*	
H9B	0.7237	0.9391	0.2721	0.028*	
H9C	0.5593	0.9556	0.2095	0.028*	
C10	0.9598 (3)	1.0150 (3)	0.13630 (15)	0.0162 (5)	
H10A	0.8951	1.1073	0.1332	0.019*	
H10B	1.0147	1.0192	0.0926	0.019*	
C11	1.0796 (3)	1.0121 (3)	0.21638 (16)	0.0202 (5)	
H11A	1.1392	1.1079	0.2231	0.024*	
H11B	1.0244	1.0054	0.2599	0.024*	
C12	1.1919 (3)	0.8801 (3)	0.22310 (19)	0.0268 (6)	
H12A	1.2463	0.8856	0.1799	0.040*	
H12B	1.1340	0.7848	0.2192	0.040*	
H12C	1.2679	0.8847	0.2745	0.040*	

### Atomic displacement parameters (Å^2^)

**Table:**

	*U*^11^	*U*^22^	*U*^33^	*U*^12^	*U*^13^	*U*^23^
Ir1	0.00843 (5)	0.00775 (5)	0.00831 (4)	0.00059 (3)	0.00184 (3)	0.00011 (3)
Cl1	0.0274 (3)	0.0203 (3)	0.0095 (3)	0.0054 (3)	0.0024 (2)	−0.0022 (2)
Cl2	0.0094 (2)	0.0104 (2)	0.0176 (3)	−0.00074 (19)	0.0014 (2)	0.0035 (2)
C1	0.0090 (10)	0.0135 (11)	0.0104 (10)	0.0028 (8)	−0.0002 (8)	0.0016 (9)
C2	0.0084 (10)	0.0128 (11)	0.0103 (10)	0.0033 (8)	0.0011 (8)	0.0022 (9)
C3	0.0118 (11)	0.0103 (10)	0.0099 (10)	0.0025 (8)	0.0024 (9)	0.0019 (9)
C4	0.0132 (11)	0.0097 (10)	0.0100 (10)	0.0026 (9)	0.0018 (9)	0.0028 (9)
C5	0.0129 (11)	0.0125 (11)	0.0083 (10)	0.0014 (9)	0.0013 (9)	0.0003 (9)
C6	0.0133 (12)	0.0180 (12)	0.0203 (13)	0.0026 (10)	0.0060 (10)	0.0000 (10)
C7	0.0189 (13)	0.0112 (11)	0.0186 (12)	0.0007 (10)	0.0031 (10)	−0.0027 (10)
C8	0.0161 (12)	0.0173 (12)	0.0156 (12)	0.0009 (10)	0.0067 (10)	0.0040 (10)
C9	0.0239 (14)	0.0199 (13)	0.0119 (11)	0.0052 (11)	0.0042 (10)	−0.0027 (10)
C10	0.0156 (12)	0.0126 (11)	0.0184 (12)	−0.0018 (9)	−0.0002 (10)	−0.0015 (10)
C11	0.0184 (13)	0.0193 (13)	0.0198 (13)	−0.0030 (10)	−0.0016 (10)	−0.0023 (11)
C12	0.0211 (14)	0.0257 (15)	0.0280 (16)	0.0005 (12)	−0.0053 (12)	−0.0009 (12)

### Geometric parameters (Å, º)

**Table:**

Ir1—Cl1	2.3924 (6)	C6—H6B	0.9800
Ir1—Cl2	2.4483 (6)	C6—H6C	0.9800
Ir1—Cl2^i^	2.4427 (6)	C7—H7A	0.9800
Ir1—C1	2.138 (2)	C7—H7B	0.9800
Ir1—C2	2.130 (2)	C7—H7C	0.9800
Ir1—C3	2.139 (2)	C8—H8A	0.9800
Ir1—C4	2.148 (2)	C8—H8B	0.9800
Ir1—C5	2.150 (2)	C8—H8C	0.9800
Cl2—Ir1^i^	2.4427 (6)	C9—H9A	0.9800
C1—C2	1.434 (3)	C9—H9B	0.9800
C1—C5	1.446 (3)	C9—H9C	0.9800
C1—C10	1.504 (3)	C10—H10A	0.9900
C2—C3	1.458 (3)	C10—H10B	0.9900
C2—C6	1.490 (3)	C10—C11	1.530 (4)
C3—C4	1.437 (3)	C11—H11A	0.9900
C3—C7	1.489 (3)	C11—H11B	0.9900
C4—C5	1.442 (3)	C11—C12	1.519 (4)
C4—C8	1.488 (3)	C12—H12A	0.9800
C5—C9	1.490 (3)	C12—H12B	0.9800
C6—H6A	0.9800	C12—H12C	0.9800
			
Cl1—Ir1—Cl2^i^	88.87 (2)	C5—C4—C8	124.5 (2)
Cl1—Ir1—Cl2	89.55 (2)	C8—C4—Ir1	126.53 (17)
Cl2^i^—Ir1—Cl2	79.89 (2)	C1—C5—Ir1	69.86 (13)
C1—Ir1—Cl1	129.38 (7)	C1—C5—C9	127.6 (2)
C1—Ir1—Cl2^i^	94.81 (6)	C4—C5—Ir1	70.30 (13)
C1—Ir1—Cl2	140.82 (6)	C4—C5—C1	107.1 (2)
C1—Ir1—C3	66.19 (9)	C4—C5—C9	125.2 (2)
C1—Ir1—C4	65.65 (9)	C9—C5—Ir1	127.85 (17)
C1—Ir1—C5	39.40 (9)	C2—C6—H6A	109.5
C2—Ir1—Cl1	97.18 (6)	C2—C6—H6B	109.5
C2—Ir1—Cl2^i^	120.22 (6)	C2—C6—H6C	109.5
C2—Ir1—Cl2	158.74 (6)	H6A—C6—H6B	109.5
C2—Ir1—C1	39.28 (9)	H6A—C6—H6C	109.5
C2—Ir1—C3	39.94 (9)	H6B—C6—H6C	109.5
C2—Ir1—C4	66.10 (9)	C3—C7—H7A	109.5
C2—Ir1—C5	66.35 (9)	C3—C7—H7B	109.5
C3—Ir1—Cl1	97.59 (6)	C3—C7—H7C	109.5
C3—Ir1—Cl2^i^	159.63 (7)	H7A—C7—H7B	109.5
C3—Ir1—Cl2	119.28 (7)	H7A—C7—H7C	109.5
C3—Ir1—C4	39.16 (8)	H7B—C7—H7C	109.5
C3—Ir1—C5	66.24 (9)	C4—C8—H8A	109.5
C4—Ir1—Cl1	130.14 (6)	C4—C8—H8B	109.5
C4—Ir1—Cl2	94.21 (6)	C4—C8—H8C	109.5
C4—Ir1—Cl2^i^	140.73 (6)	H8A—C8—H8B	109.5
C4—Ir1—C5	39.22 (9)	H8A—C8—H8C	109.5
C5—Ir1—Cl1	162.53 (6)	H8B—C8—H8C	109.5
C5—Ir1—Cl2	103.93 (7)	C5—C9—H9A	109.5
C5—Ir1—Cl2^i^	104.23 (6)	C5—C9—H9B	109.5
Ir1^i^—Cl2—Ir1	100.11 (2)	C5—C9—H9C	109.5
C2—C1—Ir1	70.05 (13)	H9A—C9—H9B	109.5
C2—C1—C5	108.8 (2)	H9A—C9—H9C	109.5
C2—C1—C10	126.8 (2)	H9B—C9—H9C	109.5
C5—C1—Ir1	70.74 (13)	C1—C10—H10A	109.3
C5—C1—C10	124.4 (2)	C1—C10—H10B	109.3
C10—C1—Ir1	125.42 (17)	C1—C10—C11	111.5 (2)
C1—C2—Ir1	70.67 (13)	H10A—C10—H10B	108.0
C1—C2—C3	107.7 (2)	C11—C10—H10A	109.3
C1—C2—C6	127.8 (2)	C11—C10—H10B	109.3
C3—C2—Ir1	70.38 (13)	C10—C11—H11A	109.1
C3—C2—C6	124.4 (2)	C10—C11—H11B	109.1
C6—C2—Ir1	127.32 (17)	H11A—C11—H11B	107.8
C2—C3—Ir1	69.68 (12)	C12—C11—C10	112.5 (2)
C2—C3—C7	125.3 (2)	C12—C11—H11A	109.1
C4—C3—Ir1	70.73 (13)	C12—C11—H11B	109.1
C4—C3—C2	107.4 (2)	C11—C12—H12A	109.5
C4—C3—C7	127.3 (2)	C11—C12—H12B	109.5
C7—C3—Ir1	127.59 (17)	C11—C12—H12C	109.5
C3—C4—Ir1	70.12 (13)	H12A—C12—H12B	109.5
C3—C4—C5	109.0 (2)	H12A—C12—H12C	109.5
C3—C4—C8	126.5 (2)	H12B—C12—H12C	109.5
C5—C4—Ir1	70.48 (13)		

Symmetry code: (i) −*x*+1, −*y*+2, −*z*.

## References

[bb1] Agilent (2011). *CrysAlis PRO* Agilent Technologies UK Ltd, Yarnton, England.

[bb2] Churchill, M. R. & Julius, S. A. (1977). *Inorg. Chem.* **16**, 1488–1494.

[bb3] Dolomanov, O. V., Bourhis, L. J., Gildea, R. J., Howard, J. A. K. & Puschmann, H. (2009). *J. Appl. Cryst.* **42**, 339–341.

[bb4] Liu, Z., Habtemariam, A., Pizarro, A. M., Fletcher, S. A., Kisova, A., Vrana, O., Salassa, L., Bruijnincx, P. C. A., Clarkson, G. J., Brabec, V. & Sadler, P. J. (2011). *J. Med. Chem.* **54**, 3011–3026. 10.1021/jm2000932 21443199

[bb5] Sheldrick, G. M. (2008). *Acta Cryst.* A**64**, 112–122. 10.1107/S0108767307043930 18156677

